# Influence of the optimization methods on neural state estimation quality of the drive system with elasticity

**DOI:** 10.1007/s00521-013-1348-4

**Published:** 2013-02-16

**Authors:** Teresa Orlowska-Kowalska, Marcin Kaminski

**Affiliations:** Institute of Electrical Machines, Drives and Measurements, Wroclaw University of Technology, Wroclaw, Poland

**Keywords:** Neural networks, State estimation, Electrical drive, Two-mass system, Training methods, Bayesian regularization, Optimal Brain Damage method

## Abstract

The paper deals with the implementation of optimized neural networks (NNs) for state variable estimation of the drive system with an elastic joint. The signals estimated by NNs are used in the control structure with a state-space controller and additional feedbacks from the shaft torque and the load speed. High estimation quality is very important for the correct operation of a closed-loop system. The precision of state variables estimation depends on the generalization properties of NNs. A short review of optimization methods of the NN is presented. Two techniques typical for regularization and pruning methods are described and tested in detail: the Bayesian regularization and the Optimal Brain Damage methods. Simulation results show good precision of both optimized neural estimators for a wide range of changes of the load speed and the load torque, not only for nominal but also changed parameters of the drive system. The simulation results are verified in a laboratory setup.

## Introduction

In most electrical drives, the elasticity of the shaft between a driving motor and a load machine must be taken into account. In order to obtain drive response to a reference signal with high dynamics, and to minimize torsional vibrations, different control methods of the drive system with elastic joint, based on control theory, like PI/PID methods, state controller-based methods, sliding-mode, and adaptive or predictive control methods [1–6] are used. All these control methods require feedbacks from different mechanical state variables of the system (load side speed, torsional torque, load torque). These mechanical variables can be measured, but only in laboratory environments. In the real drive systems, in industry, torsional or load torque can not be measured, as the torque transducer is never mounted between the driven motor and the loading machine because lack of space and generation of additional (high) cost. Similarly, the load side speed is hardly measured because lack of place for additional speed transducer and additional cabling, which is troublesome. In such a case, only estimation of those state variables is the solution for the industry conditions. This is the reason why we have to estimate the torsional torque and the load side speed of a two-mass system.

In many applications connected with electrical drives, algorithmic methods are applied for the non-measurable state variables estimation, for example, the Kalman filters [4, 5] and the Luenberger observers [6]. However, the algorithmic estimators require the mathematical model and parameter knowledge of the system, which could change during the system operation—so to obtain the good estimation quality the parameters of the state estimators must be tuned on-line (by on-line plant parameters’ identification or estimation). Alternative ways of solving this problem are estimators based on neural networks (NNs). Such estimators do not need a mathematical model and parameters of the system, only the training data are required [7–9] for the estimator design. Moreover, the generalization ability causes that neural estimators are less sensitive to parameters or measurement signals uncertainties.

However, in the case of NN applications in state variable estimation, the determination of NN structure for a specific task is one of the most important problems. This structure should be carefully chosen to obtain good estimation quality also in the case of NN input data different than those used in the training procedure. It means that a suitable generalization ability is required. Data generalization is one of the main advantages of the NN and consists in the possibility of solving a given task by a trained network in case the elements of the input vector are not taken into account in the NNs training process. In the technical literature, many methods for the improvement of the NN generalization properties are presented. It is possible to distinguish three main trends [7]:

- impact on the length of the learning process (early stopping) [10],
- application of regularisation method [11],
- modification of neural networks topology (growing or pruning) [12, 13].

Many methods for NN structure optimization are presented in the literature. Most of them require the initial choice of NN structure, and then, selected neural connections are eliminated. One of the simplest ways to choose a specific inter-node connection for elimination is the analysis of absolute values of NNs’ weights. Another method consists in checking the influence of each connection on the generalization error. In this case, the generalization errors before and after the elimination each weight factor are compared [7, 14].

Very good results are obtained with the sensitivity methods. These algorithms are based on the analysis of sensitivity of the cost function to deletion of individual connections. The most important methods in this category are the Optimal Brain Damage (OBD) [15, 16] and Optimal Brain Surgeon [17, 18] methods.

In many techniques, genetic algorithms are also applied for pruning the inter-neural connections [19].

The other solution is adding the regularization element to the cost function [17]. It consists in the modification of the objective function used in the training algorithm, which is next minimized in any iteration. In the extended form of such cost function, elements dependent on values of the inter-neural connection weights are added to the standard cost function; then, the problem of the selection of regularization parameters in the modified objective function appears. In this work, the regularization method based on the Bayesian interpretation of NNs is applied. This algorithm gives analytical formulas for automatic computation of optimal regularization parameters [20, 21].

It is reported in the literature that the Bayesian regularization method can significantly improve the quality of state variable estimation. So in this paper, the effectiveness of this method is compared with the previously used OBD method (which is rather complicated in practice [16]) for the NN state estimators of a two-mass drive system.

This paper presents neural estimators of the torsional torque and the load machine speed for a drive system with elastic joints. These neural estimators are trained with classical Levenberg–Marquardt method [7] and next they are optimized using OBD and Bayesian regularization methods. The obtained estimators are tested in the open-loop and closed-loop control structure with additional feedback adjusted suitably for damping the torsional vibration of the drive system with elastic coupling between the driven motor and the load mechanism.

The paper is divided into seven sections. After a short introduction, the mathematical model of the two-mass drive system is presented. Then, the speed control structure with a state controller and feedbacks from the motor speed, shaft torque, and the load speed are described. These two last state variables are estimated by the tested NN, and the motor speed is measured directly as well as the motor current, which form the input vectors of NN estimators. In the next part, the discussion of the NN input vector selection for the analyzed task is presented. In the forth part, the chosen methods for the improvement of the NN generalization properties are described. This paper is focused on two methods: the Bayesian regularization and the OBD method. The designed NN estimators are next implemented in the control structure and tested under simulation (section five) and experimental tests (section six). The paper is completed with short conclusions.

## Design of the state controller for a two-mass drive system

**Fig. 1:**
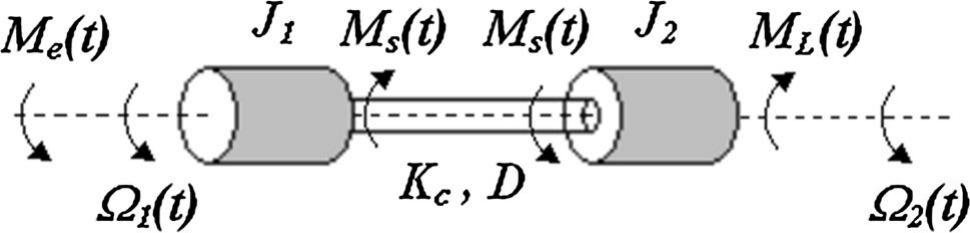
The schematic diagram of the two-mass system

The electrical drive with anelastic joint can be described by different mathematical models, depending on the exactness of the elastic shaft modeling. Usually, such drive is analyzed as a system composed of two masses connected by an elastic shaft, where the first mass represents the moment of inertia of the drive and the second mass refers to the moment of inertia of the load side (see Fig. 1). It is assumed that value of the moment of inertia of elastic shaft *J*_*c*_ is much smaller than the moments of inertia of the driving motor *J*_1_ and the load machine *J*_2_. This assumption involves the neglecting of the moment of inertia of the elastic shaft.

For the further considerations, the damping coefficient *D* of this elastic shaft is assumed as equal to zero, which leads to enlarging the influence of elasticity of the shaft on the drive system operation. Moreover, the nonlinear phenomena, like friction and backlash, are omitted; thus, the mechanical part of the considered two-mass drive can be described by the following state equation, in the per unit system, using the following notation of new state variables [2]:

1 $$ T_{1} \frac{{{\text{d}}\omega_{1} (t)}}{{{\text{d}}t}} = m_{\text{e}} (t) - m_{\text{s}} (t), $$

2 $$ T_{2} \frac{{{\text{d}}\omega_{2} (t)}}{{{\text{d}}t}} = m_{\text{s}} (t) - m_{\text{L}} (t) $$

3 $$ T_{c} \frac{{{\text{d}}m_{\text{s}} (t)}}{{{\text{d}}t}} = \omega_{1} (t) - \omega_{2} (t) $$

with:

4 $$ \omega_{1} = \frac{{\Upomega_{1} }}{{\Upomega_{\text{N}} }},\omega_{2} = \frac{{\Upomega_{2} }}{{\Upomega_{\text{N}} }},m_{\text{e}} = \frac{{M_{\text{e}} }}{{M_{\text{N}} }},m_{\text{s}} = \frac{{M_{\text{s}} }}{{M_{\text{N}} }},m_{\text{L}} = \frac{{M_{\text{L}} }}{{M_{\text{N}} }} $$

where Ω_1_, Ω_2_, Ω_N_—motor speed, load side speed, and nominal speed of the motor (rad/s), *M*_N_—nominal torque of the motor (Nm), *ω*_1_, *ω*_2_—motor and load speeds, *m*_e_, *m*_s_, *m*_L_—electromagnetic, shaft, and load torques in the per unit system.

The mechanical time constant of the motor—*T*_1_, the load machine—*T*_2_ and the stiffness time constant—*T*_*c*_ are thus given as:

5 $$ T_{1} = \frac{{\Upomega_{\text{N}} J_{1} }}{{M_{\text{N}} }},T_{2} = \frac{{\Upomega_{\text{N}} J_{2} }}{{M_{\text{N}} }},T_{C} = \frac{{M_{\text{N}} }}{{K_{c} \Upomega_{\text{N}} }}. $$

where *ω*_1_, *ω*_2_—the motor and load speeds, *m*_s_, *m*_L_—the shaft and load torques, *T*_1_, *T*_2_—the mechanical time constants of the motor and load machine, *T*_*c*_—the stiffness time constant.

**Fig. 2:**
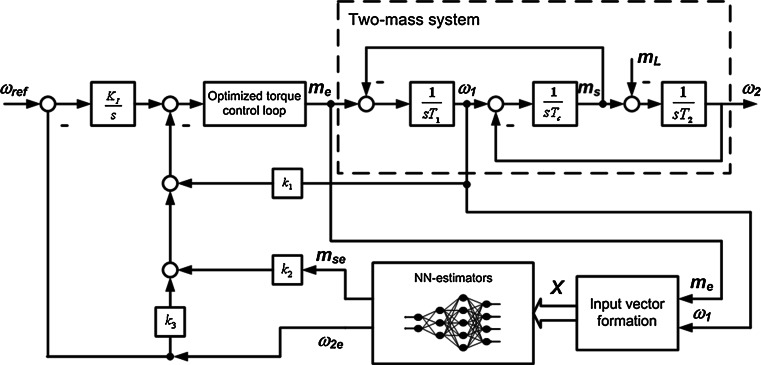
A block diagram of the control structure with a speed state controller and neural estimators for two-mass drive

The block diagram of such system with elastic connection between the motor and the load machine is shown as the part of Fig. 2 (dashed-line rectangular).

The classical cascade control structure of such drive system consists of two major control loops: the inner control loop contains the current controller, the power converter, and the motor. After optimization, the current (or torque) control loop can be replaced by the first-order inertial block with small time constant. During the design process of the speed loop, the dynamics of the torque loop is very often neglected [2]. In most cases, the PI speed controller is used in the external control loop. In this paper, the state-space controller with an integral action for steady-state error elimination is applied for the speed control of the drive system with elasticity (see Fig. 2).

Taking into account the equation for the required value of the electromagnetic torque, generated by the motor (with neglected dynamics of the torque loop and negative feedbacks from all state variables):

5 $$ m_{\text{e}} = K_{i} \int { (\omega_{r} - \omega_{2} ) {\text{d}}t} - k_{1} \omega_{1} - k_{2} m_{\text{s}} - k_{3} \omega_{2} $$

Introducing the Laplace transform for the mathematical model of the drive system (1–3) and (5), we obtain:

6 $$ T_{1} s\omega_{1} = m_{\text{e}} - m_{\text{s}} $$

7 $$ T_{2} s\omega_{2} = m_{\text{s}} - m_{\text{L}} $$

8 $$ T_{c} sm_{\text{s}} = \omega_{1} - \omega_{2} $$

9 $$ m_{e} = R(\omega_{r} - \omega_{2} ) - k_{1} \omega_{1} - k_{2} m_{s} - k_{3} \omega_{2} $$

where

10 $$ R = \frac{{K_{i} }}{s}. $$

and *s*—operator of the Laplace transform.

Including (9) in (6), the following set of equations is obtained:

11 $$ T_{1} s\omega_{1} + m_{\text{s}} = R(\omega_{r} - \omega_{2} ) - k_{1} \omega_{1} - k_{2} m_{s} - k_{3} \omega_{2} $$

12 $$ m_{\text{s}} = T_{2} s\omega_{2} + m_{\text{L}} $$

13 $$ T_{c} sm_{\text{s}} = \omega_{1} - \omega_{2} $$

Introducing (12) in (11) and (13), we obtain:

14 $$ T_{1} s\omega_{1} + T_{2} s\omega_{2} + m_{\text{L}} = R(\omega_{r} - \omega_{2} ) - k_{1} \omega_{1} - k_{2} T_{2} s\omega_{2} - k_{2} m_{\text{L}} - k_{3} \omega_{22} $$

15 $$ \omega_{1} = T_{c} T_{2} \omega_{2} s^{2} + T_{c} m_{\text{L}} s + \omega_{2} $$

then transforming this set of equations, Eq. (16) is obtained:

16 $$ \omega_{2} \left( {T_{1} T_{2} T_{c} s^{3} + T_{1} s + T_{2} s + R + k_{1} T_{c} T_{2} s^{2} + k_{1} + k_{2} T_{2} s + k_{3} } \right) = R\omega_{r} - k_{1} T_{c} sm_{\text{L}} - k_{2} m_{\text{L}} - m_{\text{L}} - T_{1} T_{c} s^{2} m_{\text{L}} , $$

which enables the determination of the transfer function of the closed-loop control system with *R* replaced by (10):

17 $$ \frac{{\omega_{2} }}{{\omega_{r} }} = \frac{{K_{i} }}{{s^{4} T_{1} T_{2} T_{c} + s^{3} k_{1} T_{c} T_{2} + s^{2} (T_{1} + T_{2} + k_{2} T_{2} ) + s(k_{1} + k_{3} ) + K_{i} }} $$

The characteristic equation of this transfer function has the following form:

18 $$ H(s) = s^{4} + s^{3} \frac{{k_{1} }}{{T_{1} }} + s^{2} \left( {\frac{1}{{T_{2} T_{c} }} + \frac{1}{{T_{1} T_{c} }} + \frac{{k_{2} }}{{T_{1} T_{c} }}} \right) + s\left( {\frac{{k_{1} }}{{T_{1} T_{2} T_{c} }} + \frac{{k_{3} }}{{T_{1} T_{2} T_{c} }}} \right) + \frac{{K_{i} }}{{T_{1} T_{2} T_{c} }}. $$

In order to calculate the expressions defining gains of the designed state controller, the characteristic equation of the closed-loop system (18) has to be compared to the reference polynomial of the same order. The following form of this polynomial was taken into account:

19 $$ H_{\text{ref}} (s) = \left( {s^{2} + 2\xi_{r} \omega_{o} s + \omega_{o}^{2} } \right)\left( {s^{2} + 2\xi_{r} \omega_{o} s + \omega_{o}^{2} } \right) = s^{4} + s^{3} (4\xi_{r} \omega_{o} ) + s^{2} \left( {2\omega_{o}^{2} + 4\xi_{r}^{2} \omega_{o}^{2} } \right) + s(4\xi_{r} \omega_{o}^{3} ) + \omega_{o}^{4} , $$

where *ξ*_*r*_, *ω*_*o*_—are the required damping factor and resonance frequency of the closed-loop system.

Comparing the elements with the same power of the Laplace operator *s*, the following expressions for the suitable gains of the state controller can be obtained:

20 $$ K_{I} = T_{1} T_{2} T_{c} \omega_{o}^{4} . $$

21 $$ k_{1} = 4T_{1} \zeta_{r} \omega_{o} , $$

22 $$ k_{2} = T_{1} T_{c} \left( {2\omega_{o}^{2} + 4\zeta_{r}^{2} \omega_{o}^{2} - \frac{1}{{T_{c} T_{2} }} - \frac{1}{{T_{c} T_{1} }}} \right), $$

23 $$ k_{3} = \omega_{o}^{2} k_{1} T_{2} T_{c} - k_{1} , $$

Feedbacks from all mechanical state variables of the two-mass system are introduced to the external control loop, so the information about the shaft torque *m*_s_, motor speed *ω*_1_, and load speed *ω*_2_ is needed. Measurement of the motor speed *ω*_1_ is simple and trouble-free, but the measurement of the shaft torque and the load speed can be difficult or expensive in the industrial practice. In this case, we can use special estimation structures based on neural networks to estimate these variables, based on easily measurable driven motor speed and current (electromagnetic torque of the driven motor is proportional to this current). So in the control structure, we will use the measured motor speed *ω*_1_ and the estimated variables, like shaft torque *m*_se_ and load speed *ω*_2e_ (see Fig. 2).

## Neural network based state variables estimators

As it was said before, the mechanical state variables required for feedback signals in the control structure of the drive system with an elastic joint have been estimated by NN-based estimators. For this research, the feed-forward NNs were selected. The previous research shows that this type of NN can give a high precision of the state variables estimation of the two-mass drive system [8], but the selection of proper NN structure is difficult and usually done by trial and error, which is a time-consuming method. To avoid this problem, we have selected some structure of NN (after a few preliminary simulation tests) and next tried to optimize this structure using two optimization methods, well known from the neural networks theory.

Starting structures are the same for both presented estimators—for the load side speed *ω*_2e_ and the shaft torque *m*_se_: {6-10-12-1}—6 inputs, 10 neurons in the 1st hidden layer, 12 neurons in the 2nd hidden layer, and 1 neuron in the output. For the hidden layers, the nonlinear tangensoidal activation functions are applied. The linear activation function is selected as the output function of the considered neural estimators.

The proper selection of elements of the input vector of neural network is very important for correct realization of the required task. The selection of input elements in the design process of neural estimators should take into account the properties of NNs and practical aspects of the analyzed implementation. It should be noticed that the expansion of the input vector of NN can influence the structure of the net, in result it influences on its practical implementation (e.g., using FPGA—for the time of calculation and consumption of resources). At the same time, in the case of an expanded input vector, results of NN calculation can improve slightly, or—on the contrary—they can be even worse. From the engineering point of view, signals included in the NN input vector should be selected carefully to fulfill the following conditions:

- give an important information about changes of the state variables of the process
- they should be easily measured in the real system.

According to these requirements, the input signals of the neural networks in our case are the motor speed *ω*_1_ and the electromagnetic torque *m*_e_ (or stator current) of the driven motor.

In the presented application of neural estimation, MLNN (multi-layer neural networks) were implemented. It should be noted that the NNs analyzed in the described application are static systems; they do not have internal feedbacks or memories. On the other hand, the presented application is focused on dynamical signals of the drive system, quickly changing in time. Therefore, to take into account the dynamics of the processed signals and to obtain better quality of the state variable estimation, the input vector of MLNN was extended with the delayed samples of input variables (motor speed and electromagnetic torque). So the form of the assumed input vector is described by the following equation:

24 $$ {\mathbf{X}} = [\omega_{1} (k),\omega_{1} (k - 1),\omega_{1} (k - 2),m_{\text{e}} (k),m_{\text{e}} (k - 1),m_{\text{e}} (k - 2)] $$

The number of historical samples was selected experimentally. For the analyzed data, type of NNs and number of iterations in the training process, the best results were obtained for input vector described by expression (24). Tests of estimators without delayed samples of input signals in the processed vector lead to much worse results. On the other hand, increasing the number of historical samples [in comparison with Eq. (24)] only slightly influences the precision of estimation and is not necessary from the point of view of practical implementation.

The Levenberg–Marquardt (LM) learning algorithm is used to train the NN state estimators. The value of each weight coefficient is adjusted according to LM, while the error backpropagation (EBP) is used to calculate the Jacobian matrix of the cost function with respect to the weight values. The updating rules of NN weights **w** are presented below:

25 $$ \Updelta {\mathbf{w}} = - ({\mathbf{J}}^{T} {\mathbf{J}} + \eta {\mathbf{I}})^{ - 1} {\mathbf{J}}^{T} {\mathbf{e}} $$

where **J**—Jacobian matrix of the cost function *E* with respect to the weight values, *η*—learning factor, **I**—identity matrix, **e**—difference between target output of the training data and the network output.

Next, the previously selected structure of NN estimators was optimized using the Bayesian regularization and OBD methods. The effectiveness of these methods in the described task has been compared and evaluated.

## Optimization methods used for neural estimators

### Bayesian regularization method

The neural networks training process can be defined as a minimization of the objective function. In the considered case, the analyzed cost function is described by a following equation:

26 $$ F = \beta E_{D} + \alpha E_{W} $$

where element *E*_*D*_ is a sum of squares of NN calculation errors for each input sample, and *E*_*W*_ is an additional regularization term presented below:

27 $$ E_{D} = \sum\limits_{j = 1}^{M} {(d_{j} - y_{j} )^{2} } , $$

28 $$ E_{W} = \sum\limits_{i = 1}^{W} {w_{i}^{2} } , $$

where *d*_*j*_—desired output values; *y*_*j*_—actual output values of the neuron; *M*—dimension of the vector **d**, *w*_*i*_—weights; *W*—the total number of weight and biases in the network.

In relation to the objective function (26), the problem of selecting parameters *α* and *β* appears. The regularization parameters describe the influence of suitable terms *E*_*R*_ and *E*_*D*_ on the cost function. The first one decides about NN exactness in respect to the training data, and the second one enforces the smoothness of NN output [20, 21]. If *α* is relatively significant in comparison with *β*, the training error is smaller and the effect is like in a classical algorithm. In the other case, the training process gives smaller weights and leads to a smoother network output. Therefore, the optimal values for those factors are extremely important to achieve good estimation quality. In many cases, these parameters can be chosen using cross-validation techniques, but this procedure is time consuming. In the Bayesian interpretation of NNs, the optimization of inter-neural weights corresponds to the increase of probability:

29 $$ P({\mathbf{w}}|{\mathbf{D}},\alpha ,\beta ,A) = \frac{{P({\mathbf{D}}|{\mathbf{w}},\beta ,A)P({\mathbf{w}}|\alpha ,A)}}{{P({\mathbf{D}}|\alpha ,\beta ,A)}} $$

where **w**—weight coefficient vector, **D**—training data, *A*—structure of the neural network, *P*(**D**|*α,β,A*)—normalization element, *P*(**w**|*α,A*)—describes the information on the weights’ values before introducing the training data, *P*(**D**|**w** *,β,A*)—probability of obtaining the established response of the NN for suitable inputs, depending on parameters of the network.

Under the assumption that noise in the input data (measurements) used in the process of NN training is a Gaussian and the probability of weight distribution is also a Gaussian, suitable elements in Eq. (29) are described by the following formulas:

30 $$ P({\mathbf{D}}|{\mathbf{w}},\beta ,A) = \frac{1}{{Z_{D} (\beta )}}\exp ( - \beta E_{D} ) $$

and

31 $$ P({\mathbf{w}}|\alpha ,A) = \frac{1}{{Z_{W} (\alpha )}}\exp ( - \alpha E_{W} ), $$

where

32)-(33 $$ Z_{D} (\beta ) = \left( {\frac{\pi }{\beta }} \right)^{\frac{M}{2}} \;{\text{and}}\;Z_{W} (\alpha ) = \left( {\frac{\pi }{\alpha }} \right)^{\frac{W}{2}} , $$

thus, we obtain:

34 $$ P({\mathbf{w}}|{\mathbf{D}},\alpha ,\beta ,A) = \frac{{\frac{1}{{Z_{D} (\beta )}}\frac{1}{{Z_{R} (\alpha )}}\exp ( - (\alpha E_{W} + \beta E_{D} ))}}{{P({\mathbf{D}}|\alpha ,\beta ,A)}} $$

For the optimization of *α* and *β* parameters in the objective function, the following equation is taken into account:

35 $$ P(\alpha ,\beta |{\mathbf{D}},A) = \frac{{P({\mathbf{D}}|\alpha ,\beta ,A)P(\alpha ,\beta |A)}}{{P({\mathbf{D}}|A)}}. $$

Under the assumption that distribution of regularization coefficients *α* and *β* is uniform, maximal values of the probability *P*(*α*,*β*|**D**,*A*) are obtained for the biggest values of the element *P*(**D**|*α*,*β*,*A*). Probability *P*(**D**|*A*) is independent of the required parameters. After suitable transformations [20, 21], equations describing *α* and *β* parameters for the minimum of the objective function are obtained:

36 $$ \alpha = \frac{\gamma }{{2E_{W} ({\mathbf{w}}_{\text{MP}} )}} $$

and

37 $$ \beta = \frac{M - \gamma }{{2E_{D} ({\mathbf{w}}_{\text{MP}} )}}, $$

where

38 $$ \gamma = W - 2\alpha \;{\text{trace}}({\mathbf{H}})^{ - 1} . $$

and **w**_MP_—minimum point of the objective function, **H**—hessian matrix of the cost function.

The parameter *γ* means an effective number of parameters of the NN; however, *W* is a number of all parameters in the NN.

### Optimal Brain Damage method

The neural networks training leads to the minimization of the cost function defined as a mean square error between estimated and real value.

The cost function, for *p*-elements learning vector, is described in the following way:

39 $$ E = \frac{1}{2}\sum\limits_{j = 1}^{p} {\sum\limits_{i = 1}^{M} {(d_{i} (j) - y_{i} (j))^{2} } } . $$

Differentiability and continuity of the cost function (39) make possible to use the gradient methods for its minimization. The first step in this method is an expansion of the cost function into Taylor series around the actual solution:

40 $$ \Updelta E = \sum\limits_{i} {g_{i} \Updelta w_{i} } + \frac{1}{2}\left[ {\sum\limits_{i} {h_{ii} [\Updelta w_{ii} ]^{2} } + \sum\limits_{i \ne j} {h_{ij} \Updelta w_{i} \Updelta w_{j} } } \right] + {\rm O}\left( {\left\| {\Updelta w} \right\|^{3} } \right) $$

where Δ*w*_*i*_-changes of *i*-th weight;

41 $$ g_{i} = \frac{\partial E}{{\partial w_{i} }}; $$

42 $$ h_{ij} = \frac{{\partial^{2} E}}{{\partial w_{i} \partial w_{j} }}. $$

In the OBD algorithm, the weight coefficients are eliminated after full training of the net, so we can assume that elements related to the gradient are equal zero and skip them in Eq. (40). The hessian matrix is diagonally dominant, which makes it possible to include only diagonal elements *h*_*ii*_ of this matrix in the presented algorithm. The quadratic approximation assumes that the cost function is quadratic, so the third element in the Eq. (40) can be neglected. Following the above assumptions, the saliency coefficient is described by the following relation [15]:

43 $$ S_{i} = \Updelta E = \frac{1}{2}\sum\limits_{i} {h_{ii} [\Updelta w_{ii} ]^{2} } $$

These coefficients give the information about influence of the respective connections in NN on the training process. The weights with the smallest saliency parameter are eliminated. The algorithm of OBD method is thus presented as follows:

1. Choice of reasonable topology of neural network.
2. Full training of the net.
3. Computing diagonal elements of the *h*_*ii*_.
4. Evaluation of the *saliency* parameters *S*_*i*_ for every weights coefficients.
5. Deleting the elements with the smallest saliency.
6. If weights connections were deleted, go back to the second point with reduced topology of neural network.

## Simulation results

The NN estimators are tested in the control structure presented in Fig. 2. The main parameters of the drive system are as follows: *T*_1_ = *T*_2_ = 203 ms and *T*_*c*_ = 2.6 ms. The assumed values of resonant frequency and the damping factor of the speed closed loop of the drive system are, respectively: *ω*_*o*_ = 45 s^−1^ and *ξ*_*r*_ = 0.7. For disturbance reduction, which are caused by high dynamics of inputs signals and measurement noise, the low-pass filters are used with time constant *T* = 5 ms.

**Fig. 3:**
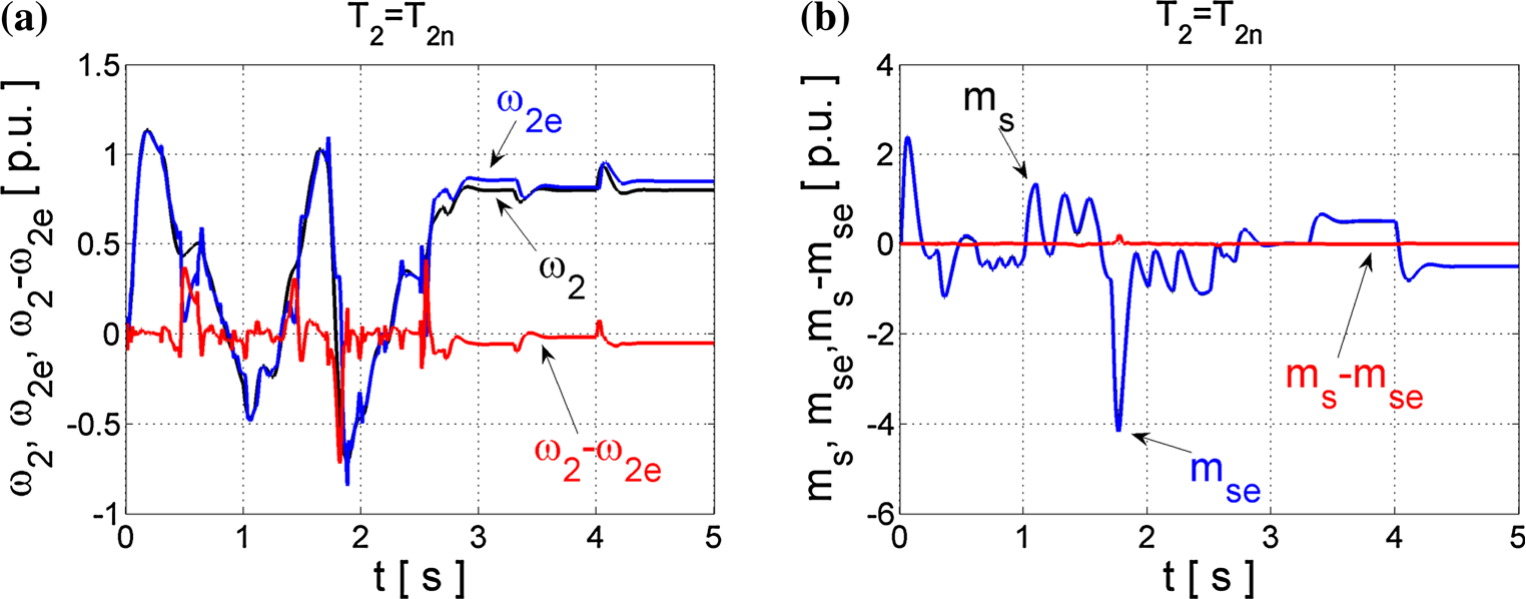
Transients of the real and estimated load speed *ω*_2_ (**a**) and torsional torque *m*_s_ (**b**) and their estimation errors obtained for NN trained with LM algorithm and tested in an open loop

The first results are presented for NNs trained with the Levenberg–Marquardt algorithm, without any additional techniques. The neural estimators are tested first in the open control loop, which means that control structure of the two-mass system is based on state variables obtained directly from the drive mathematical model, and signals estimated by the designed NNs are not used in this structure. The obtained results are shown in Fig. 3.

In order to evaluate the quality of estimation of the load machine speed *ω*_2e_ and shaft torque *m*_se_, the estimation errors of NNs are calculated, using the following formula:

44 $$ {\text{Err}} = \frac{{\sum\nolimits_{i = 1}^{n} {\left| {x_{i} - \hat{x}_{i} } \right|} }}{N} \times 100 $$

where *x*_*i*_—real value, $\hat{x}_{i}$—estimated value, *N*—number of samples.

The estimation errors (average error per sample) calculated for transients presented in Fig. 3, are, respectively, 5.77 for the load speed and 0.63 for the shaft torque.

**Fig. 4:**
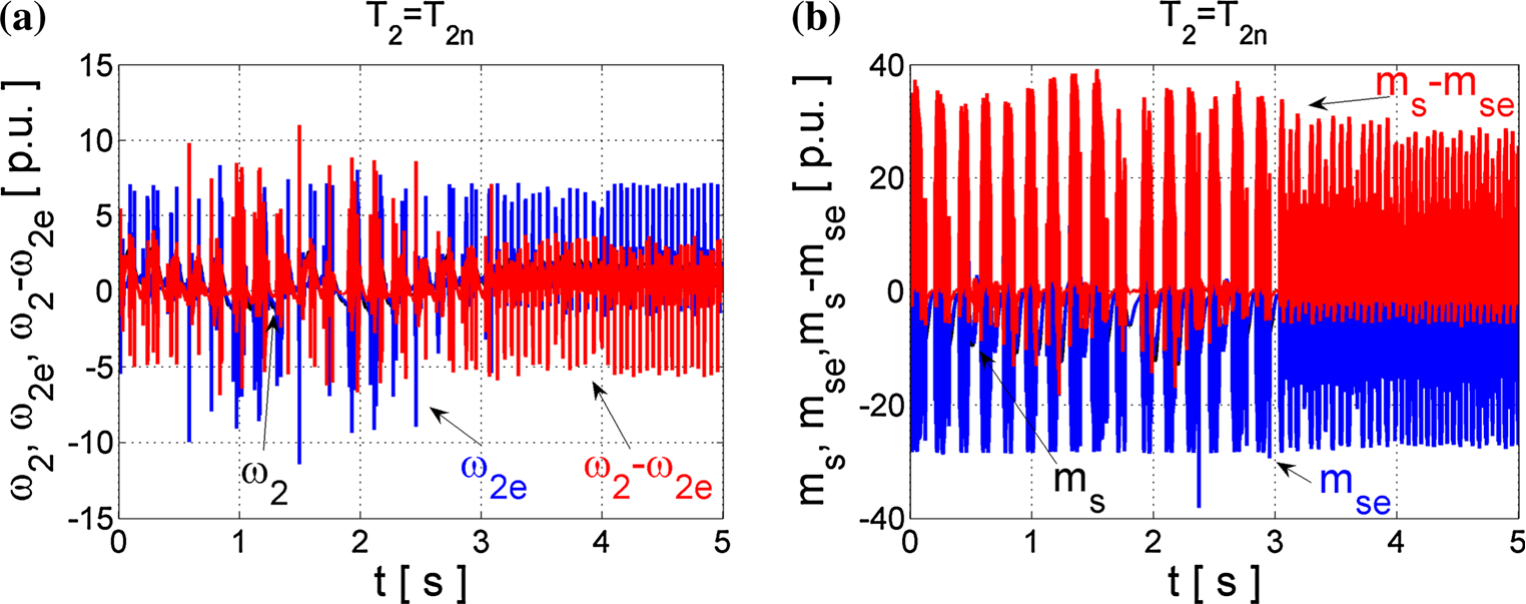
Transients of the real and estimated load speed *ω*_2_ (**a**) and torsional torque *m*_s_ (**b**), and their estimation errors obtained from NNs trained with LM algorithm and tested in the closed-loop system

Next, the estimated signals were introduced into the control structure and obtained results are demonstrated in Fig. 4. As can be seen from those transients, neural estimators prepared using the Leveneberg–Marquardt algorithm and next tested in the closed control loop failed.

The NNs are not considered during the designing process of the control structure. The coefficient values (20–23) in the suitable feedback loops are calculated with the assumption that we have the exact knowledge of feedback signals, so in the case of estimated variables, these “ideal” coefficients intensify dynamical estimation errors and thus quite significant interferences appear. Oscillations of state variables are excited in the closed-loop structure, so the proper control is impossible. These phenomena may cause damages of coupling elements between the motor and load machine. So the high quality of state variables estimation is necessary for the correct operation of the closed-loop drive system. Thus, the optimization methods for NNs are introduced.

**Fig. 5:**
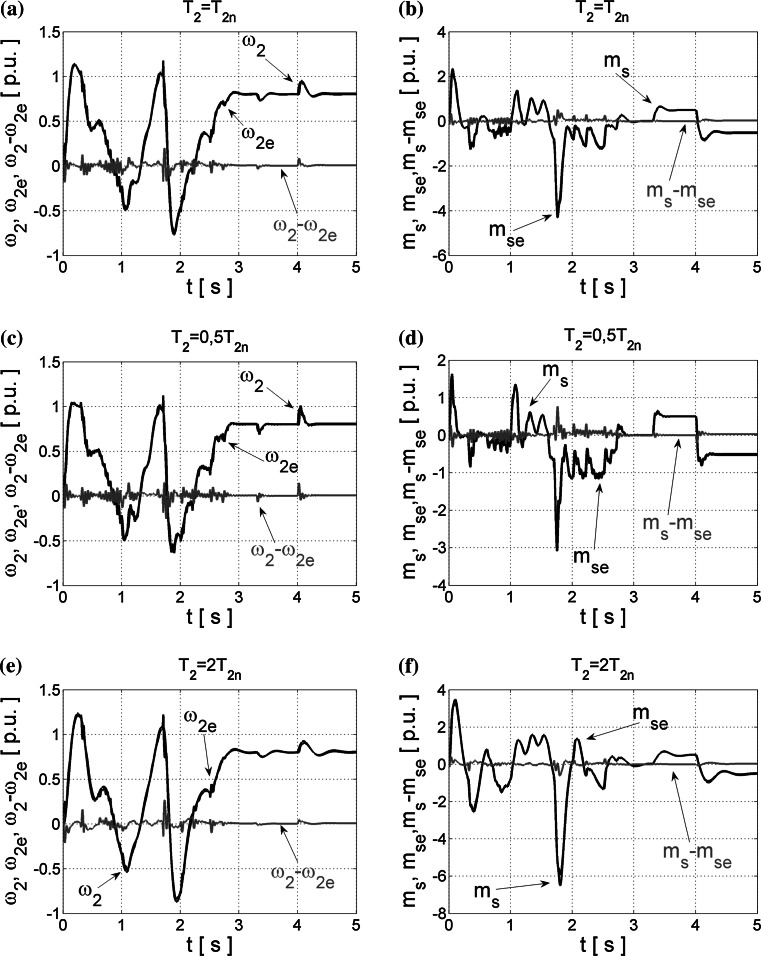
Transients of the real and estimated load speed *ω*_2_ (**a**, **c**, **e**) and torsional torque *m*_s_ (**b**, **d**, **f**) and their estimation errors obtained from NN trained with LM algorithm and the Bayesian regularization, and tested in the closed loop for different values of *T*_2_ time constant

First, the application of Bayesian regularization in neural estimators is tested, and operation of the obtained NNs implemented in the closed-loop system is presented in Fig. 5, also in the case of changeable load side time constant *T*_2_.

The obtained results are very good, and the usage of the modified cost function (26–28) during the training procedure of NN can eliminate too big weight coefficients of the designed neural estimators and thus prevent oscillations appearing previously in the closed-loop operation. The correct operation of the designed estimators in the closed control loop can be assured even for changeable values of the load mechanism time constant *T*_2_ (Fig. 5c–f).

**Fig. 6:**
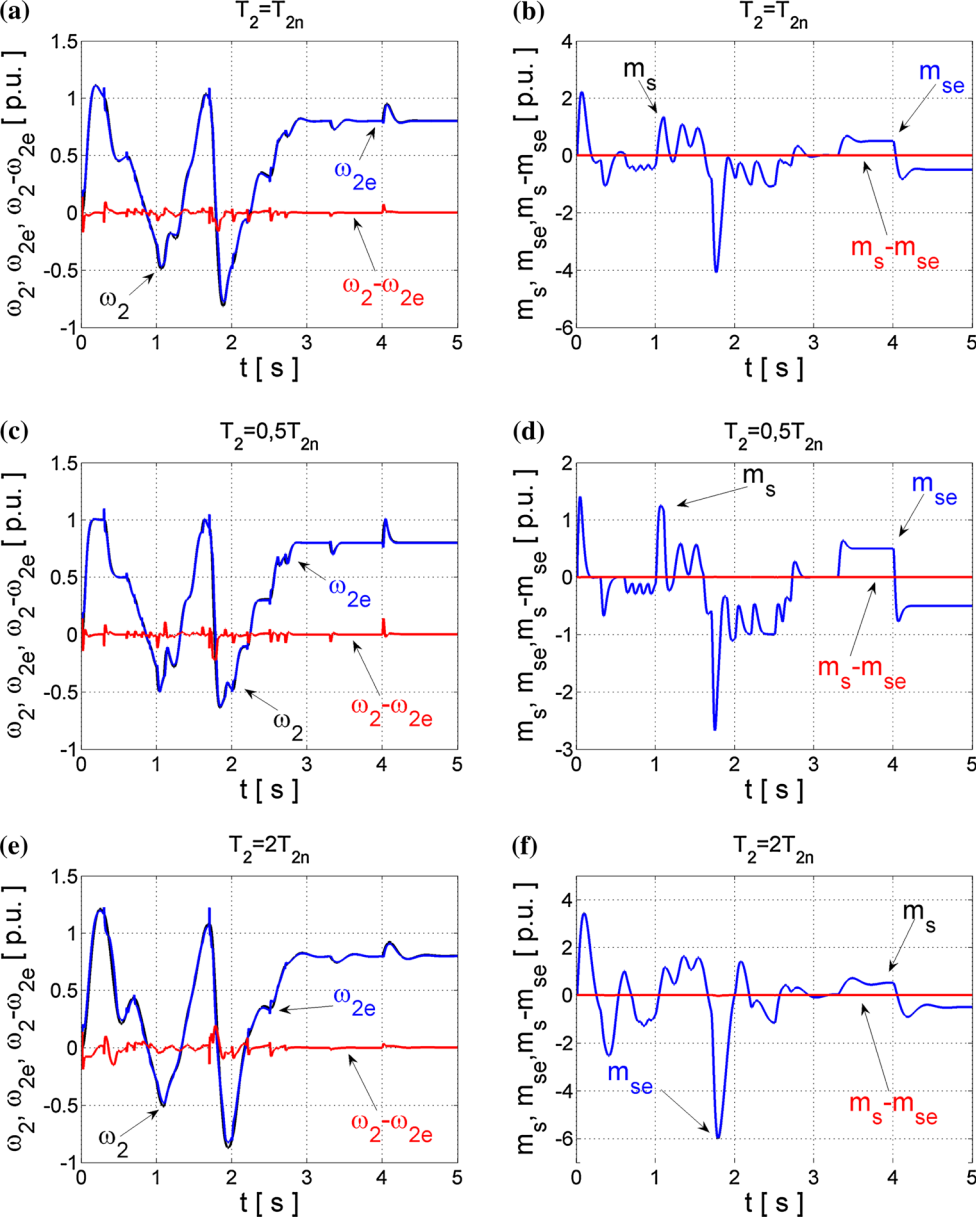
Transients of the real and estimated load speed *ω*_2_ (**a**, **c**, **e**) and torsional torque *m*_s_ (**b**, **d**, **f**) and their estimation errors obtained from NNs trained error with LM algorithm and the OBD method, tested in closed loop for different values of *T*_2_ time constant

However, the best quality of the state estimation is achieved for NN structures optimized with the OBD method. The obtained results of closed-loop operation are demonstrated in Fig. 6.

**Fig. 7:**
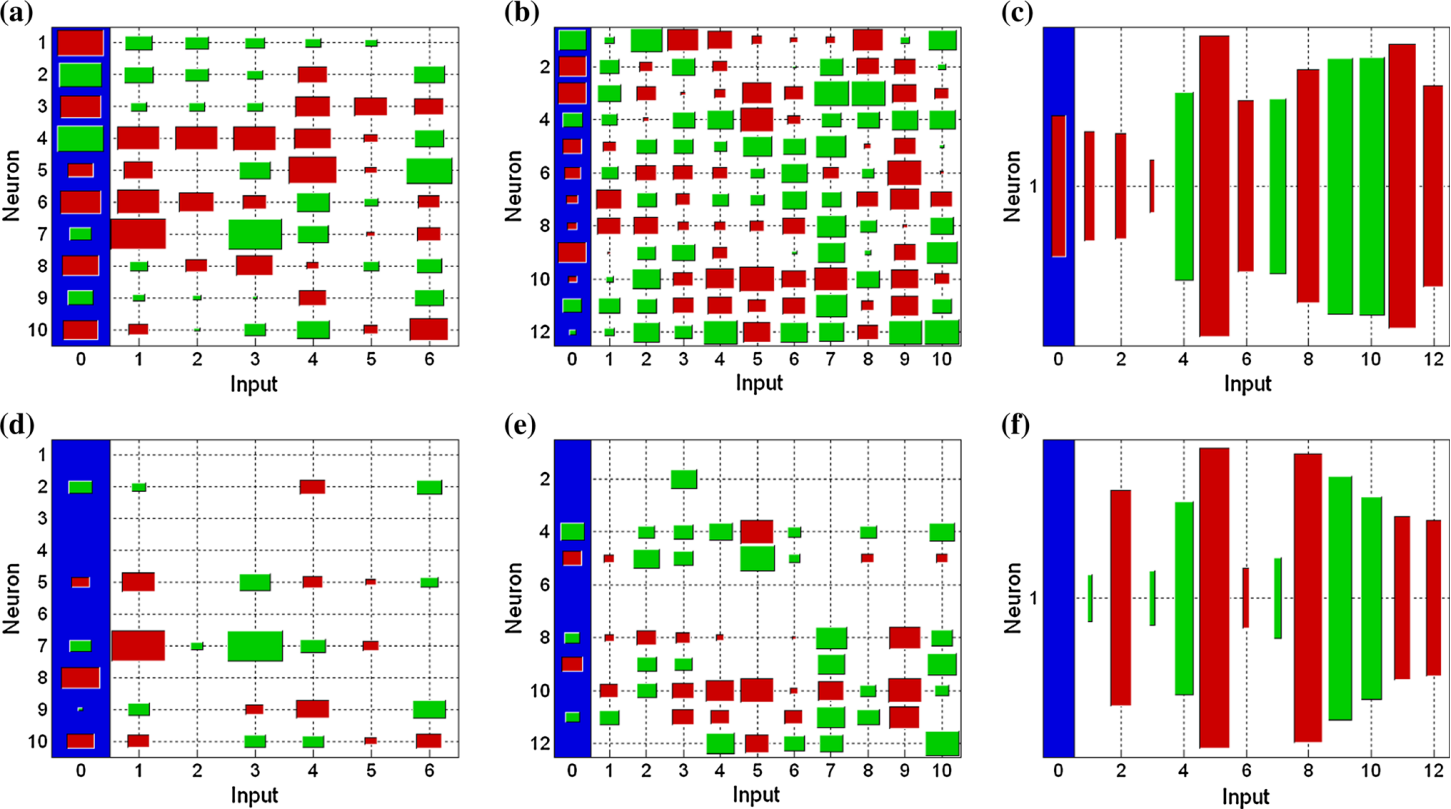
Hinton diagrams for the load speed estimator illustrating weight values between input and first hidden layer (**a**, **d**), between two hidden layers (**b**, **e**) and between second hidden and output layers (**c**, **f**) before OBD method (**a**–**c**) and after weight elimination (**d**–**f**)

Neural estimators optimized with this method can calculate precisely the suitable state variables of the two-mass drive system even in the presence of *T*_2_ changes. It is important that changes of the time constant of the load machine are not taken into account during the training process in both tested cases. The neural estimators have the initial structure containing 215 synaptic coefficients. After OBD method applied for the shaft torque estimator, 80 synaptic connections are deleted and for the load speed estimator, 140 connections are eliminated, respectively. The decision about stopping the optimization process is based on the analysis of the estimation error. The examples of the Hinton diagrams for the load speed estimators are presented in Fig. 7. The Hinton diagrams visualize matrices of bias and weights values. Each value is represented by a rectangle, which size is associated with the weight magnitude, and each color indicates the sign (a positive—red, a negative—green). The OBD algorithm eliminates individual inter-neural connections; however, there are neurons completely eliminated after this optimization process (Fig. 7d, e).

In the Table 1, the comparison of the estimation errors, calculated according to (44) for both tested methods, is shown. Both described methods enable the preparation of neural estimators which give the correct results after implementation in the closed-loop structure of the two-mass drive system.

**Table 1:** Errors for neural estimators tested in the closed loop for changes of the *T*_2_ time constant

Method	*T*_2_ time constant		
	*T*_2_ = *T*_2N_	*T*_2_ = 0.5*T*_2N_	*T*_2_ = 2*T*_2N_
Estimator *m*_s_			
Bayesian regularization	4.60	5.05	5.05
OBD	0.05	0.05	0.11
Estimator *ω*_2_			
Bayesian regularization	2.16	2.44	2.19
OBD	1.28	1.28	2.12

The OBD method can give better results, but for this method, higher computational power is required and the training process is much slower than for the Bayesian regularization method. The pruning methods are also important when the hardware implementation of the neural estimators is considered. It is possible to reduce the structure of the NN which leads to the simplification of the realization algorithm and in result to save the hardware resources (e.g., in the case of FPGA implementation).

## Experimental results

**Fig. 8:**
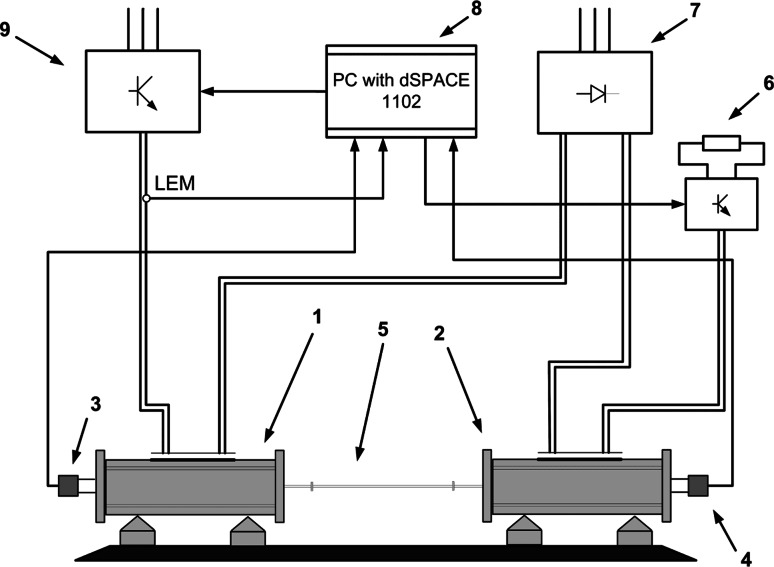
Schematic diagram of the experimental setup, where *1*—motor machine, *2*—load machine, *3*,*4*—encoders, *5*—shaft, *6*—resistor, *7*—rectifier, *8*—control structure, *9*—power converter

The tested drive system with an elastic joint is emulated with two DC machines (0.5 kW each) connected by an elastic shaft (a steel shaft of 5 mm diameter and 600 mm length). The stiffness of the connection depends on the shaft diameter. The motor is fed by a power converter. The control algorithm and neural state estimators are implemented in DSP placed in the dSPACE 1102 card. The load machine in the drive system is also controlled using the DSP (see Fig. 8). Basic parameters of the drive system are presented in the Table 2.

**Table 2:** Parameters of the two-mass system

Parameter	Value	Unit
Power	500	W
Nominal motor voltage	220	V
Nominal speed	1,450	rev/min
Motor mechanical time constant	0.203	s
Load mechanical time constant	0.203	s
Shaft length	600	mm
Shaft diameter	6	mm
Stiffness time constant	0.0026	s

**Fig. 9:**
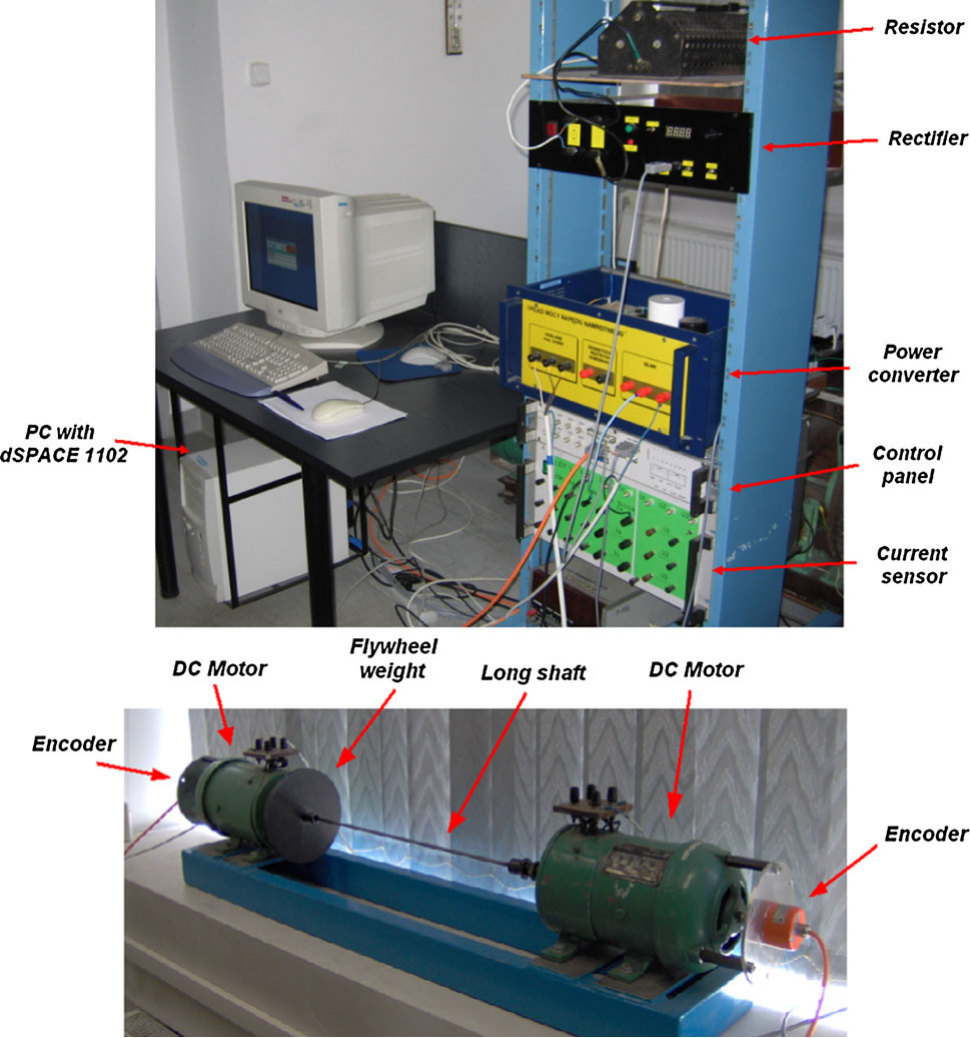
Laboratory test bench

The speeds of both DC machines are measured by incremental encoders (36 000 pulses per rotation); however, the measurement of the loading machine is used only for comparison with the estimated value. In the laboratory setup the LEM sensors for current measurements are implemented. There is no shaft torque sensor in the laboratory setup. Therefore, in order to check the estimated shaft torque shape, the Kalman filter is applied [4, 22]. In Fig. 9, pictures of the laboratory test bench are presented.

**Fig. 10:**
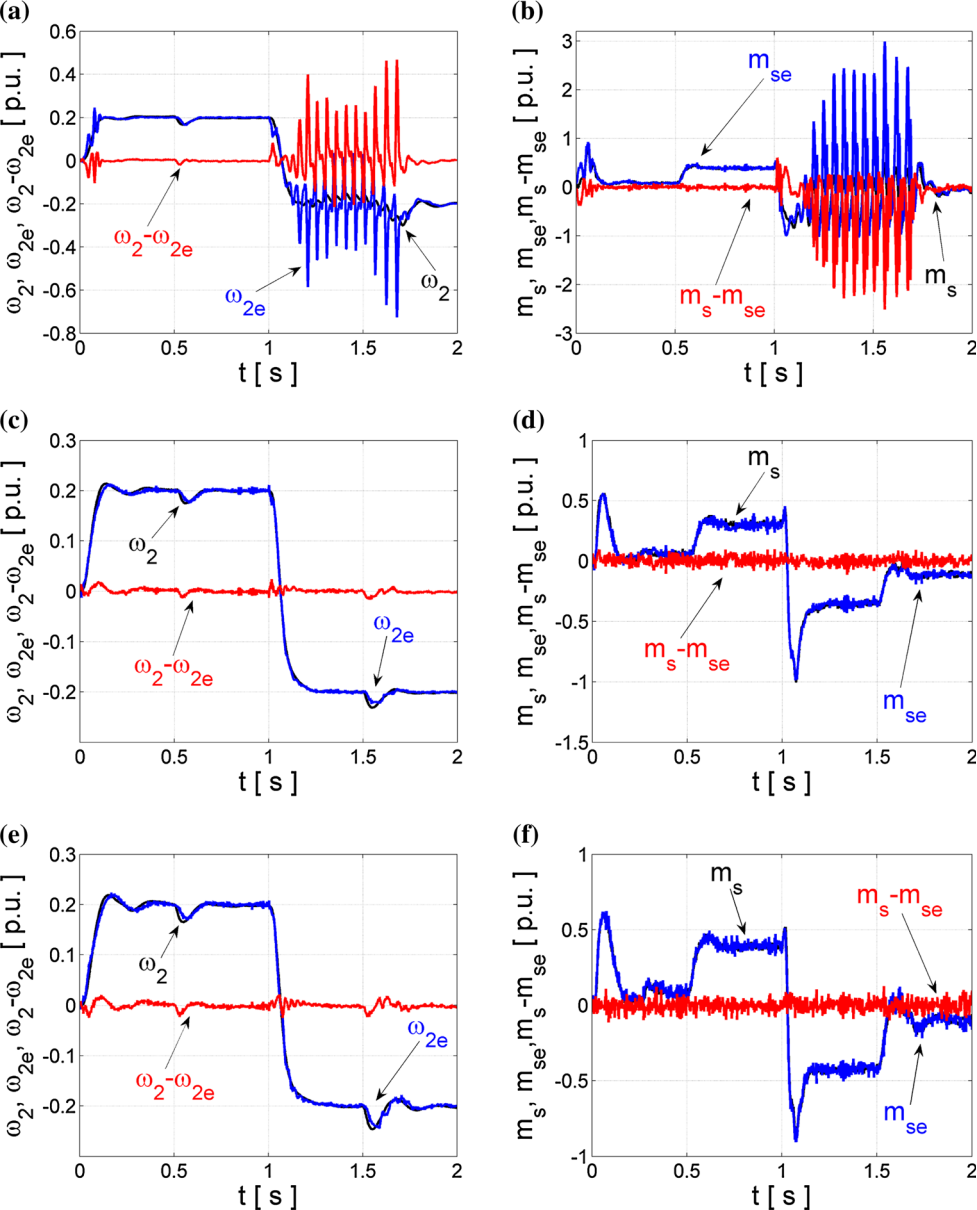
Experimental transients (closed-loop operation for nominal *T*_2_ value) of the real and estimated load speed *ω*_2_ (**a**, **c**, **e**) and torsional torque *m*_s_ (**b**, **d**, **f**) and their estimation errors obtained for NN trained with LM algorithm (**a**, **b**), and after the Bayesian regularization (**c**, **d**), and the OBD method (**e**, **f**)

Exemplary transients of the state estimation in the closed-loop drive structure, obtained before and after NNs optimization, are presented in the Fig. 10.

**Fig. 11:**
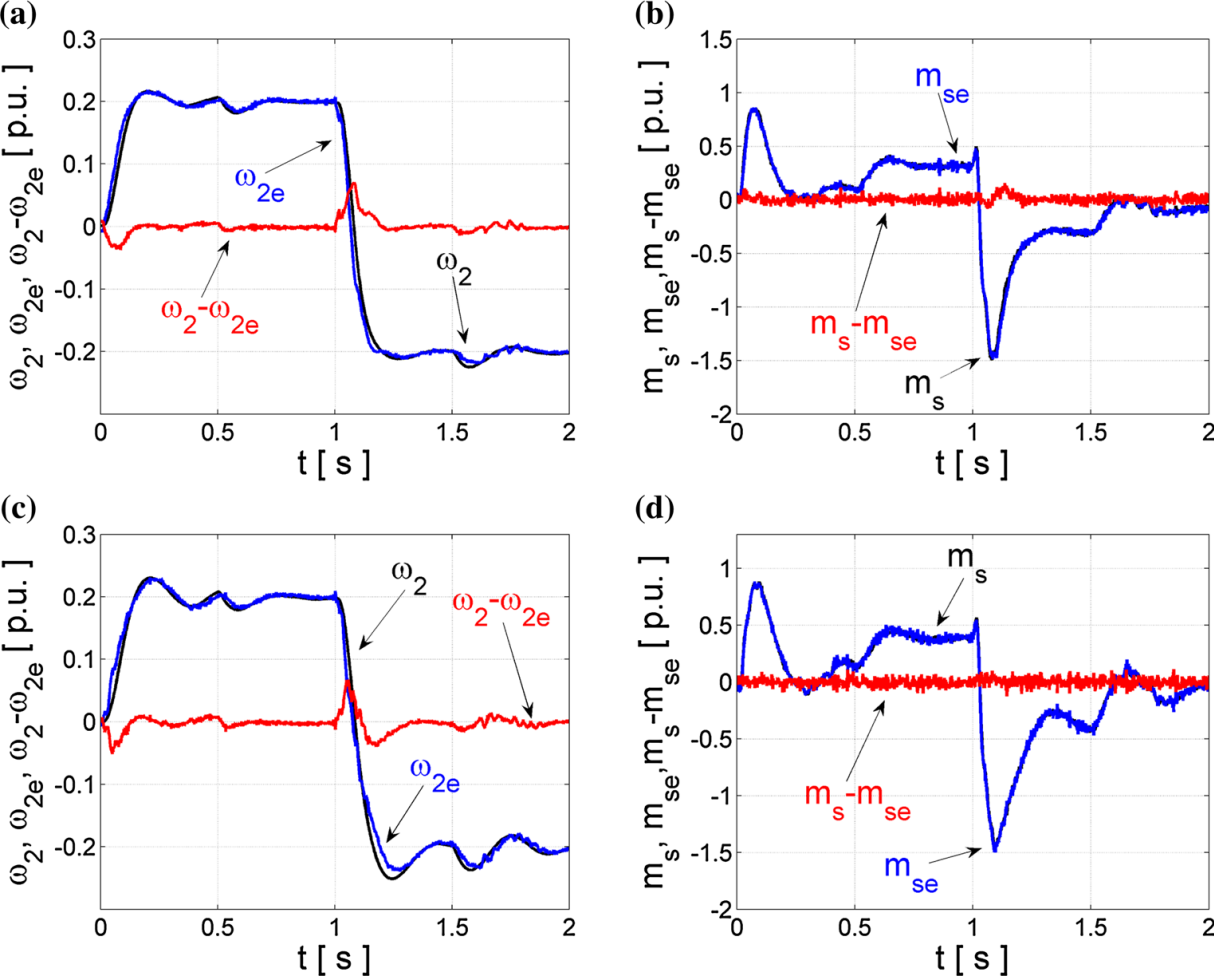
Experimental transients (closed-loop operation for *T*_2_ = 2*T*_2N_) of the real and estimated load speed *ω*_2_ (**a**, **c**) and torsional torque *m*_s_ (**b**, **d**) and their estimation errors obtained for NN after optimization with the Bayesian regularization (**a**, **b**), and the OBD method (**c**, **d**)

The tests are realized for the reference speed that equals 20 % of its nominal value; after one second, the reverse operation of the drive system is forced. In the period *t* ∈ (0.5–1.5)s, the nominal load torque is applied. Next, this load is taken off. Similarly to the simulation, neural estimators trained with Levenberg–Marquardt algorithm are generating noises and instability of the closed-loop drive system operation (Fig. 10a, b). After optimization with the described algorithms, both estimators work properly. The estimation errors (44) for both optimization algorithms are similar in the case of experimental tests: for the NN optimized with Bayesian regularization, the load speed error is 0.37 and the torsional torque error is 2.77. The inaccuracy of the load speed and torsional torque reconstruction for neural estimator designed with the OBD method are, respectively, 0.49 and 2.99. Additional tests for twice bigger *T*_2_ time constant were conducted. The results are presented in the Fig. 11.

As in simulation tests, the changes of the two-mass system parameter are not taken into account during neural estimator training, also coefficients in the control structure are calculated for nominal value of *T*_2_. Estimation error of the load speed is equal 0.67 in the case of the Bayesian regularization method and 0.83 for the OBD method. Torsional torque reconstruction using neural estimators optimized with Bayesian regularization method presents error equal to 2.76 and after implementation of the OBD method is 2.95. Comparing changes of the errors for different values of *T*_2_ in the drive system similar values can be observed. The conclusion is that obtained neural estimators are robust against changes of the tested drive parameter.

## Conclusion

Application of neural estimators in the drive system with elastic coupling enables very good estimation quality. Neural estimators do not require the knowledge of mathematical model parameters on the contrary to the algorithmic methods of state variable estimation. Disturbances from the estimated signals connected as additional feedbacks in the two-mass drive control structure can lead to the speed oscillation or even problems with system instability. So the good quality of the estimation is very important. After the implementation of Bayesian regularization or OBD, the obtained precision of calculations in NN is much better. Presented estimators are also robust to changes of the mechanical parameters of the drive, like the load side time constant *T*_2_. The OBD method can give slightly better results, but it should be noticed that for this method higher computational power is required and the training process is much slower than for the Bayesian regularization method. Thus, this last method can be recommended for practical implementation. Correct work of the designed estimators was confirmed not only by simulations but also by experiments in the real drive system in the laboratory.
